# Dopant-Free π-Conjugated Hole Transport Materials for Highly Stable and Efficient Perovskite Solar Cells

**DOI:** 10.3389/fchem.2021.664504

**Published:** 2021-03-18

**Authors:** Zhifeng Deng, Shuaiwei Cui, Kaichang Kou, Dongxu Liang, Xin Shi, Jinhui Liu

**Affiliations:** ^1^School of Chemistry and Chemical Engineering, Northwestern Polytechnical University (NWPU), Xi'an, China; ^2^National and Local Joint Engineering Laboratory for Slag Comprehensive Utilization and Environmental Technology, School of Materials Science and Engineering, Shaanxi University of Technology, Hanzhong, China; ^3^Key Laboratory of Rubber-Plastic of Ministry of Education (QUST), School of Polymer Science and Engineering, Qingdao University of Science and Technology, Qingdao, China

**Keywords:** π-conjugated materials, perovskite solar cells, hole transfer material, dopant-free, molecular design

## Abstract

Current high-efficiency hybrid perovskite solar cells (PSCs) have been fabricated with doped hole transfer material (HTM), which has shown short-term stability. Doping applied in HTMs for PSCs can enhance the hole mobility and PSCs' power conversion efficiency, while the stability of PSCs will be significantly decreased due to inherent hygroscopic properties and chemical incompatibility. Development of dopant-free HTM with high hole mobility is a challenge and of utmost importance. In this review, a series of selected and typical π-conjugated dopant-free hole transport materials, mainly regarding small molecules, are reviewed, which could consequently help to further design high-performance dopant-free HTMs. In addition, an outline of the molecular design concept and also the perspective of ideal dopant-free HTMs were explored.

## Introduction

Currently, the state-of-the-art perovskite solar cells with conventional *n*-*i*-*p* structures utilize 2,2′,7,7′-tetrakis(*N, N*′-di-*p*-methoxyphenlamine)-9,9-spirobifluorene (spiro-OMeTAD) or poly-triarylamine (PTTA) as HTMs. However, the HTMs like spiro-OMeTAD and PTAA suffer from low mobility (< 1 × 10^−5^ cm^2^ V^−1^ s^−1^) and limited conductivity (< 3 × 10^−7^ S cm^−1^) (Vivo et al., 2017). These materials thus need hazardous dopants to increase the hole mobility and conductivity; these can include such dopants as bis(trifluoromethane sulfonyl)imide lithium salt (LiTFSI), which may also facilitate device degradation due to the sophisticated oxidation process associated with undesired ion migration and chemical interaction with underlying perovskite layer (Yu and Sun, 2015; Abi Ghanem et al., 2019). In addition, such dopants would make the device prone to hygroscopicity. Considering their drawbacks, over the last few years, a wide range of novel HTMs have been proposed as alternatives by the state-of-the-art organic π-conjugated small molecule and polymer; however, only a few candidates have reached similar initial performance compared to the state-of-the-art HTMs. Unfortunately, those candidates still require high amounts of dopants to operate (Yu and Sun, 2015; Vivo et al., 2017). Even though a few operational dopant-free materials could be obtained, the power conversion efficiency (PCE) still lacks behind the state-of-the-art HTMs (Huang et al., 2016; Liu et al., 2016; Yun et al., 2016; Zhao et al., 2016; Lee et al., 2017). Normally, HTMs would not require an additional doping process if they exhibit hole mobility up to 10^−4^-10^−3^ cm^2^ V^−1^ s^−1^ and have matched energy levers. Therefore, the development of high-performance dopant-free HTMs is highly desirable and of utmost importance. Molecular design strategies, such as large π-conjugation and strong π-π stacking (Paek et al., 2017), strong planarity (Yun et al., 2016), surface passivation (Cao et al., 2015), introducing functional groups, and novel chemical structures, have been considered to be the most feasible and effective methods mainly used for designing dopant-free HTMs that possess better intermolecular interactions to ensure sufficient hole mobility. In this article, dopant-free π-conjugated small molecules applied to PSCs are reviewed.

## Large π-Conjugation and Strong π-π Stacking

**Figure 1:**
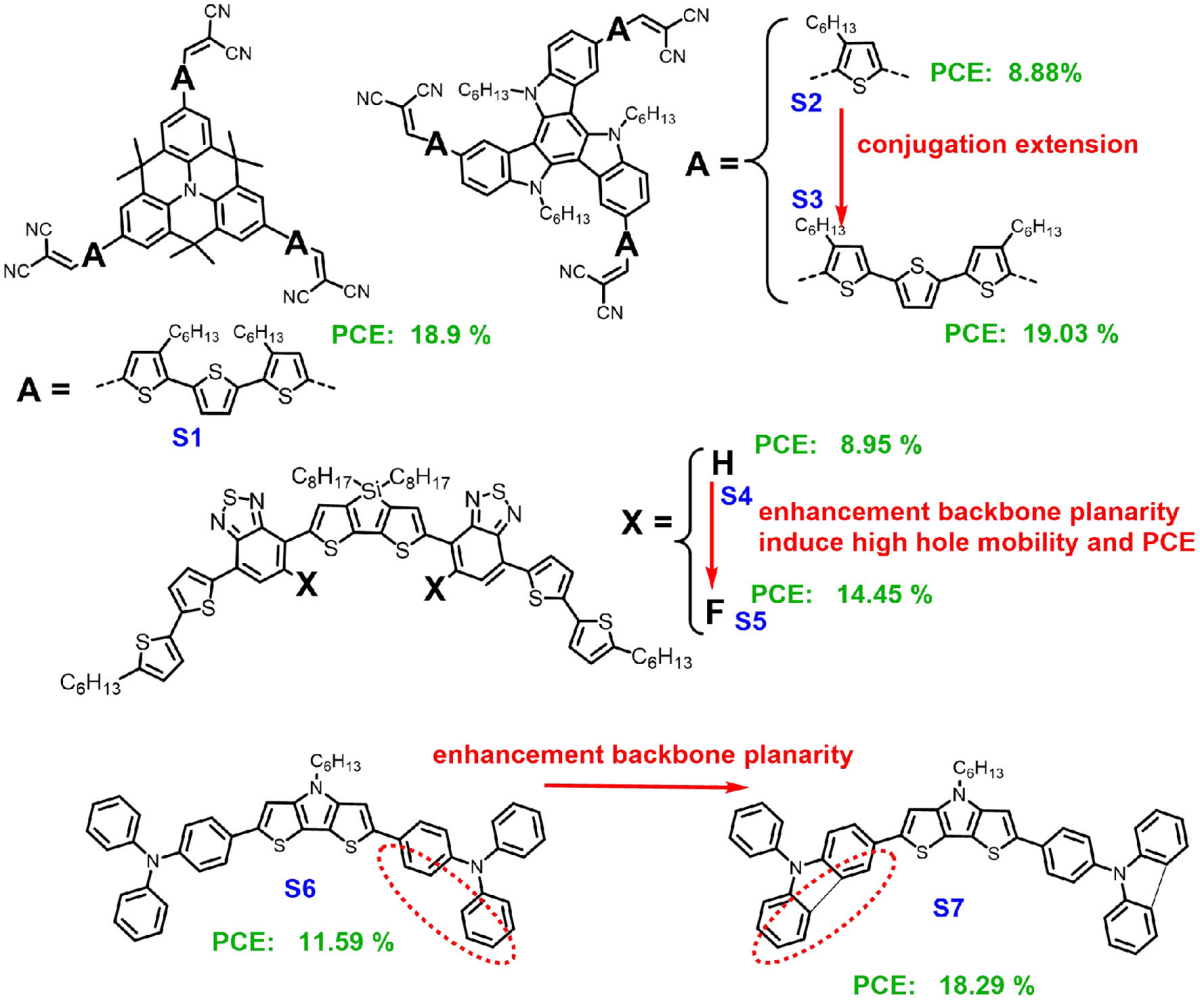
Chemical structures of small molecules S1–S7 for dopant-free HTMs.

Dopant-free HTMs are divided into small molecules and polymers. Compared to polymers, small organic molecular HTMs have received great attention due to their high efficiency and simple synthetic schemes (Zhang et al., 2017a). Simultaneously, they were also shown to have better intra-molecular hole transfer mobility due to better packing. In general, the molecular structure of organic HTMs consists of peripheral donors and central cores, such as pyrene (Ge et al., 2018), fluorene (Rakstys et al., 2017b), carbazole (Chen et al., 2017), benzothiophene (Zimmermann et al., 2017), and indacenodithienothiophene (Liu et al., 2017). The studies on dopant-free organic HTM are quite rare, among which most reported molecules contain thiophene or thiophene derivatives as the donor units or π-conjugated bridge. Very recently, K. Nazeeruddin and co-authors using rigid quinolizino acridine (FA), which is widely used in molecular semiconductors due to its planarity and strong π-π stacking, as the central donor core, tri-thiophene as the π-conjugated bridge, and malenonitrile as the strongest electron acceptor to stabilize the highest occupied molecular orbital (HOMO) energy level, which built a branch oligomer (S1, Figure 1) and showed PCE of 18.9% and a maximum power output that was collected after 1300 h that remained at 65% of its initial value (Paek et al., 2017). In addition, this group introduced thiophene or tri-thiophene as branches into the same core and synthesized two oligomers (S2 and S3, Figure 1), which were also used as HTMs to fabricate PSCs (Rakstys et al., 2017a). The results showed that π-extension would significantly enhance the PCE of the PSCs from 8.88 to 19.03%.

## Strong Planarity

Except for π-conjugation extension, the planarity of a molecular backbone is crucial for the molecular design concept of high-performance dopant-free HTMs. J. Son and co-authors introduced fluorine atoms to the molecules for adjusting the planarity of the backbone and the molecular packing through sulfur–fluorine interactions, which significantly increased the hole mobility and the PCE from 8.95% (S4) to 14.45% (S5) (Yun et al., 2016). Very recently, through a comprehensive study on the deliberate molecular design and modifications of electron donors, correlations between the planarity of HTMs backbone and performance of PSC were reported by Liang's group (Li et al., 2020). In this work, the dithieno[3,2-b:2′,3′-d]pyrrole (DTP) both end substituted by the twisted triphenylamine as the donor (S6) or the capped-by-planar-*N*-phenyl-carbazole donor were synthesized (S7). We used two small molecules, S6 and S7, as dopant-free HTMs to fabricate PSCs, which showed PCEs with 11.59% (S6) and 18.29% (S7). The chemical structure between triphenylamine and the *N*-phenyl-carazole are quite similar except for an additional chemical bond connecting between the two phenyl rings of the triphenylamine; however, the PCE of the S7-based device showed a value almost 60% higher than the one based on S6. The significantly different performance between these two small molecule-based PSCs could be ascribed to S7 with an improved planarity backbone, resulting in a good hole transfer ability.

## Surface Passivation (Interfacial Interaction)

**Figure 2:**
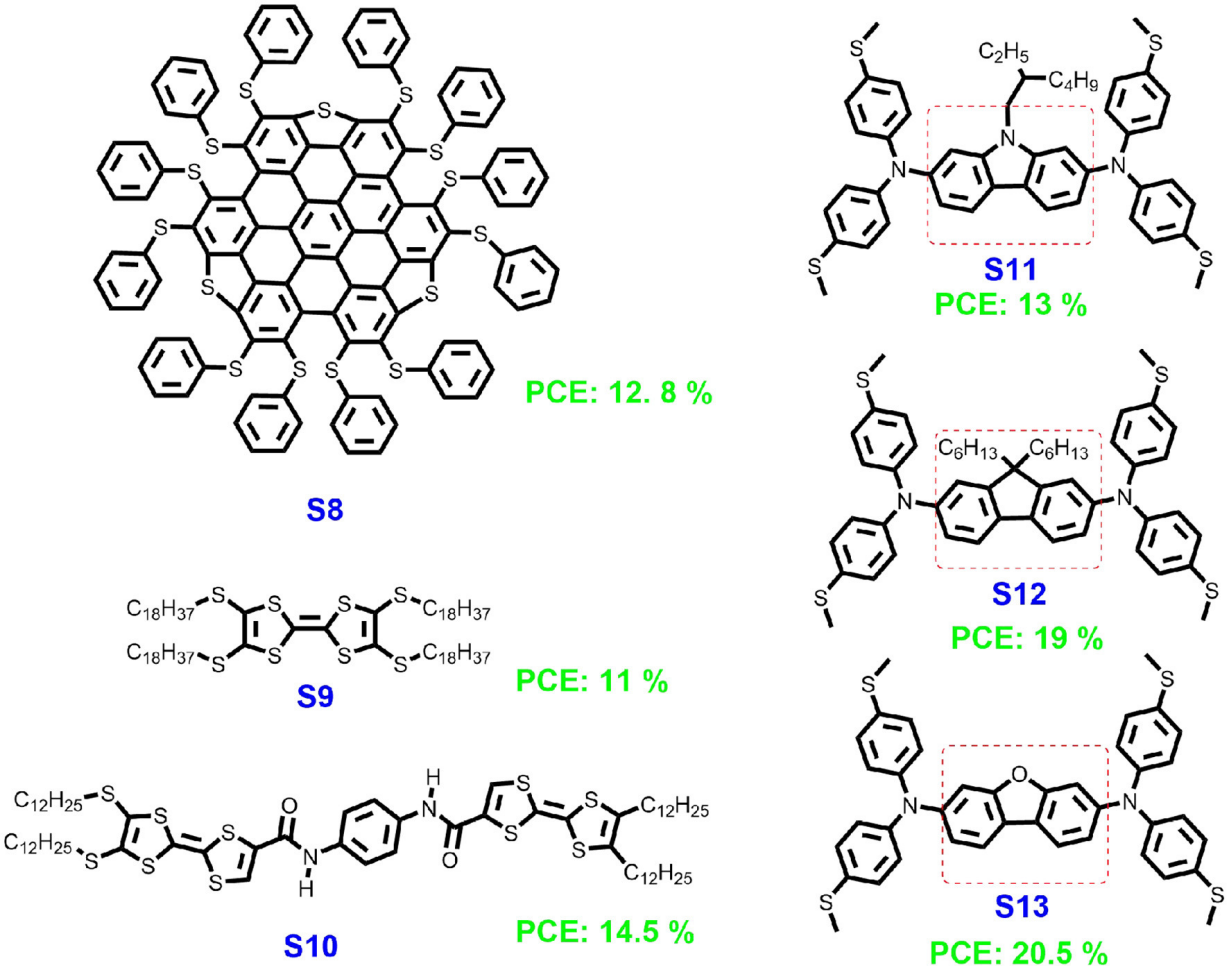
Chemical structures of small molecules S8–S13 for dopant-free HTMs.

In a photovoltaic device, after light absorption, the generated charge carriers (electron/hole) need to be transported through the perovskite layer and collected at the adjacent charge selective interfaces (Zhang et al., 2017b). Each of these steps plays a key role in the high performance of PSCs. Among them, the quite important (and mostly neglected) part is the hole transfer between the interface of HTM and the perovskite layer. To the best of our knowledge, most reported articles focus on the modification of the HTM's HOMO/LUMO energy levels but not on the inter-layer interaction (Yu and Sun, 2015; Vivo et al., 2017). Compatible HOMO/LUMO energy levels for HTMs will be beneficial for the hole transfer and to block the electron transfer from the perovskite layer to the HTM layer to enable the maximum achievable open-circuit voltage. The good HTM should not only have a compatible HOMO energy level but should also provide intimate inter-layer interaction since the interface interaction would help to rapidly extract holes from the perovskite layer and transfer them to the HTM layer, resulting in a low energy loss and reduced recombination of charge carriers at the interface. Zheng's group reported that a thiolated nanographene perthiolated trisulfur-annulated hexa-peri-hexabenzocoronene (S8, Figure 2), as the HTM in the pristine form in PSCs (Cao et al., 2015). The thiol groups at the periphery form Pb-S coordination-bonds at the interface between perovskite and HTM, which was investigated by the infrared spectra. The tight binding of S8 helps to rapidly extract charge from perovskite, resulting in a low energy loss at the interface and PCE of 12.8%. The performance is readily improved by doping with graphene sheets into tri-sulfurannulated hexa-*peri*-hexabenzocoronene (TSHBC), which formed a novel functionalized nanographene that could enhance the hole transporting property within HTM. In addition, M. Zhan and co-workers reported on the introduction of an interaction layer between the perovskite and HTM layers, which could act as Lewis bases and interact with Pb atoms to form trap states that greatly passivate the defects on the surface of the perovskite layer (Zhang et al., 2017b). This technique could significantly enhance the efficiency and stability of the PSCs. Except for sulfur atoms, silicon and nitrogen atoms can also provide lone pair electrons to form Pb-N or Pb-Si coordination-bonds through the formation of Lewis adduct between the under-coordinated Pb atoms at the HTM-perovskite interface. A deeper understanding of the relationship between Pb-S/Pb-N/Pb-S interaction at perovskite/HTM interface and the PSC's performance should be established in the near future.

## Introducing Functional Group

Research into organic electronics revealed that not only the composition of the organic material but also subtle changes in the material structure (the way how the molecules are stacked) can noticeably alter its bulk properties (Yao et al., 2016). One of the reasons is that the charge transport in conjugated materials is strongly affected by their structures. For totally disordered organic small molecular films, charge mobility is low, while mobility significantly increases if the materials exhibit self-assembling properties that can be exploited to generate ordered structures (Zhang et al., 2018, 2020). Jian et al. designed and synthesized tetrathia-fulvalene derivative (S9, Figure 2) and used it as dopant-free HTM, which showed a PCE of 11.03% in 2014, fill factor (FF) of 64%, and open-circuit voltage (*V*_oc_) of 0.86 V (Liu et al., 2014). A few years later, Islam's group introduced two amide units into these small molecules (S10) (Kaneko et al., 2019). In S10, intermolecular hydrogen bonds could be formed between amino and the carbonyl groups of the neighboring two molecules in the solid states. Using this small molecule to fabricate the PSCs, a PCE of 14.5% with the *V*_oc_ of 1.11 V and the FF of 66% were achieved. The increased PSCs performance could be ascribed to the following facts: (i) hydrogen bonding formation between the adjacent molecules, which could improve the inter-molecular hole transfer mobility; and (ii) the exiting multi-nitrogen and oxygen atoms could form surface passivation between the HTMs layer and the perovskite layer, which is beneficial for the hole transfer between the two layers.

## Materials With Novel Chemical Structures

For high-performance dopant-free HTMs for PSCs, the development of novel chemical structures are important. For commercial applications, it is a challenge to find suitable and low-cost HTMs in PSCs. In 2017, Ding's group designed a novel HTM based on carbazole and *N, N*-di-*p*-methylthiophenylamine, which is named S11 (Figure 2) (Xu et al., 2017). The dopant-free S11-based planar *p-i-n* perovskite solar cells exhibit a high PCE of 13.05% with a *V*_oc_ of 1.03 V and FF of 58.23%. Later, the same group modified the structure using 9,9-dihexyl-*9H*-fluorene instead of the carbazole core obtained S12 (Zhang et al., 2019). Using S12 to fabricated the PSCs showed significantly improved performance with a higher PCE of 19.06%, *V*_oc_ of 1.07 V, and FF of 79.27%. In 2020, the authors further optimized the HTMs structures and changed the core into dibenzo[b,d]furan, resulting in S13. The performance of the PSC-based S13 even further improved with a PCE up to 20.51%, *V*_oc_ of 1.07 V, and FF of 80.48% (Quan et al., 2020). This investigation indicates that the molecular design is quite important for high-performance HTMs, and developing novel structures is thus crucial.

## Conclusions and Outlook

Research regarding HTMs for PSCs is crucial and could significantly improve the performance of perovskite solar cell devices in terms of power conversion efficiency, open-circuit voltage, fill factor, etc. Furthermore, due to the high conductivity and hole transfer mobility, HTMs do not need dopant in the HTM layer. The cost of the HTMs is thus decreased; meanwhile, the lifetimes of the PSCs are extended. This review revealed that the ideal HTMs of small molecules could be achieved with high conductivity and hole transfer mobility with low cost and suitable energy levels. To obtain such ideal dopant-free HTMs, several molecular design concepts are summarized in this review, such as enlarging the π-conjugation system, increasing the planarity of molecular backbone, introducing the functional atoms/groups to achieve interface interaction between the HTMs layer and the perovskite layer, and, resulting in self-assembly within the HTMs layer, the development molecules with novel chemical structures. HTMs play a key role in the high performance of PSCs. Future research should focus on the combination of part or all of the abovementioned design concepts in a single molecule to develop desirable HTMs.

## Author Contributions

ZD and SC prepared the manuscript. DL and XS helped to prepare the references and revise the manuscript. KK revised the manuscript. ZD and JL supervised the whole work. All authors discussed and commented on the paper.

## Conflict of Interest

The authors declare that the research was conducted in the absence of any commercial or financial relationships that could be construed as a potential conflict of interest. The handling Editor declared a past co-authorship with one of the authors ZD.
