# Supplementary figures and images for: Dopant-Free π-Conjugated Hole Transport Materials for Highly Stable and Efficient Perovskite Solar Cells
Source: Front Chem. 2021 Mar 18;9:664504. doi: 10.3389/fchem.2021.664504 (PMC8012559; doi:10.3389/fchem.2021.664504)

**Toc:**


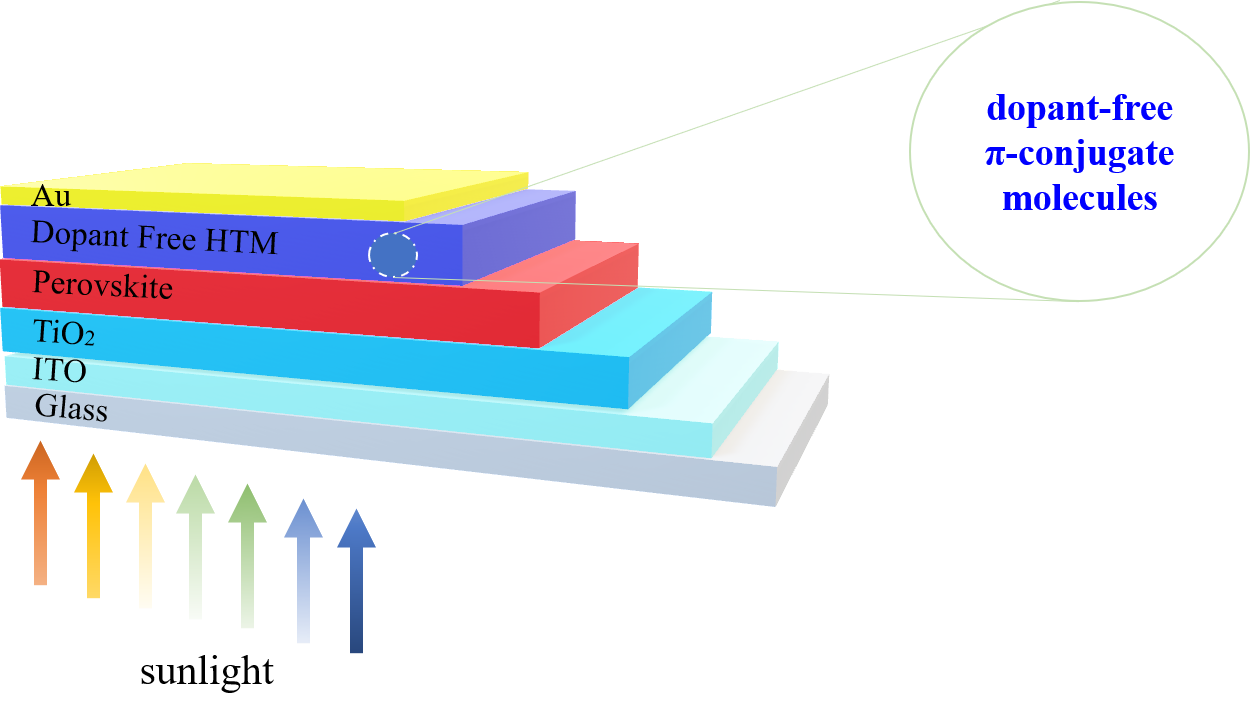

Supplement: Supplementary file 1 [file Data_Sheet_1.docx]
